# In Vitro Antimicrobial and Antibiofilm Properties and Bioaccessibility after Oral Digestion of Chemically Characterized Extracts Obtained from *Cistus* × *incanus* L., *Scutellaria lateriflora* L., and Their Combination

**DOI:** 10.3390/foods12091826

**Published:** 2023-04-28

**Authors:** Hammad Ullah, Alessandro Di Minno, Anna De Filippis, Eduardo Sommella, Daniele Giuseppe Buccato, Lorenza Francesca De Lellis, Hesham R. El-Seedi, Shaden A. M. Khalifa, Roberto Piccinocchi, Massimiliano Galdiero, Pietro Campiglia, Maria Daglia

**Affiliations:** 1 Department of Pharmacy, University of Napoli Federico II, Via D. Montesano 49, 80131 Naples, NA, Italy; hammad.ullah@unina.it (H.U.); alessandro.diminno@unina.it (A.D.M.); d.buccato@gmail.com (D.G.B.); lo.delellis2@libero.it (L.F.D.L.); 2 CEINGE-BiotecnologieAvanzate, Via Gaetano Salvatore 486, 80145 Naples, NA, Italy; 3 Department of Experimental Medicine, Section of Microbiology and Clinical Microbiology, University of Campania “L. Vanvitelli”, Via De Crecchio, 7, 80138 Naples, NA, Italy; anna.defilippis@unicampania.it (A.D.F.); massimiliano.galdiero@unicampania.it (M.G.); 4 Department of Pharmacy, University of Salerno, 84084 Fisciano, SA, Italy; esommella@unisa.it (E.S.); pcampiglia@unisa.it (P.C.); 5 Pharmacognosy Group, Department of Pharmaceutical Biosciences, Uppsala University, Biomedical Centre, SE-751 24 Uppsala, Sweden; hesham.el-seedi@farmbio.uu.se; 6 International Research Center for Food Nutrition and Safety, Jiangsu University, Zhenjiang 212013, China; 7 Department of Molecular Biosciences, The Wenner-Gren Institute, Stockholm University, S-106 91 Stockholm, Sweden; shaden.khalifa@regionstockholm.se; 8 Level 1 Medical Director Anaesthesia and Resuscitation A. U. O. Luigi Vanvitelli, Via Santa Maria di Costantinopoli, 80138 Naples, NA, Italy; roberto.piccinocchi@policliniconapoli.it; 9 UOC of Virology and Microbiology, University Hospital of Campania “Luigi Vanvitelli”, 80138 Naples, NA, Italy; 10 European Biomedical Research Institute of Salerno, Via De Renzi 50, 84125 Salerno, SA, Italy

**Keywords:** *Cistus* × *incanus* L., *Scutellarialateriflora* L., oral health, gingivitis, *Porphyromonas* *gingivalis*

## Abstract

Periodontal diseases are oral inflammatory diseases ranging from gingivitis to chronic periodontitis. *Porphyromonas gingivalis* is one of the major pathogens responsible for severe and chronic periodontitis. Plant extracts with antimicrobial activity could be considered possible alternatives to chlorhexidine, an antiseptic substance used in oral hygiene thatcan cause bacteria resistance. Here, two commercial extracts obtained from *Cistus* × *incanus* L. and *Scutellaria lateriflora* L. were chemically characterized usingUltra-High-Performance Liquid Chromatography (UHPLC) coupled with a Q-Exactive Hybrid Quadrupole Orbitrap Mass Spectrometer. The extracts were studied for their bioaccessibility after simulated in vitro oral digestion, their antimicrobial activity against *P. gingivalis*, their protective effects against cellular invasion by *P. gingivalis*, and their antibiofilm activity. The extracts were found to contain very complex mixtures of polyphenols, which were quite stable after in vitro simulated oral digestion and demonstrated mild, dose-dependent inhibitory activity against *P. gingivalis* growth. This activity increased with the combination of the two extracts. Moreover, the combination of the extracts induced a reduction in *P. gingivalis* HaCaT invasiveness, and the reduction in biofilm came to around 80%. In conclusion, a combination of *C. incanus* and *S. lateriflora* showed promising effects useful in the treatment of gingivitis.

## 1. Introduction

Periodontal diseases include a range of chronic inflammatory conditions affecting the gingiva, bones, and tooth ligaments. They generally begin with plaque-induced gingivitis, initiated by bacteria embedded within the plaque near the gum line. Untreated gingivitis may progress to the loss of gingiva, bones, and ligaments, which may lead to chronic periodontitis, the ultimate result of which is the initiation of deep periodontal pockets (a hallmark of the disease) and tooth loss [1,2]. It is widely accepted that the etiology of periodontal disease is driven by several factors, including host immunity, environmental factors, and periodontal pathogens, i.e., *Porphyromonas gingivalis*, *Treponema denticola*, and *Tannerella forsythia*, which form the so-called “red complex”. Accumulation of supragingival and subgingival polymicrobial biofilm communities induces a persistent host immune response within the periodontium [3]. The inflammation process can be reversed with the removal of the biofilm and, as a result, inflammation can be limited to the gingival epithelium and connective tissues. Nevertheless, if the persistent accumulation of biofilm leads to an irreversible inflammatory process, the disease progresses from gingivitis to periodontitis with the involvement of deeper periodontal tissues (i.e., the deepening of the gingival crevice, the destruction of periodontal ligaments, and alveolar bone loss) [4]. The outgrowth of periodontal pathogens further encourages this inflammatory cascade, and proteinaceous byproducts of tissue production (i.e., amino acids, collagen breakdown products, iron, and heme) reinforce the growth of periodontal pathogens, further increasing the inflammatory cascade [5,6,7]. 

Periodontal diseases are more prevalent in adults but may also occur in younger subjects (children and adolescents), where the amount of tissue destruction is usually commensurate with dental plaque levels, host defenses, and associated risk factors [8]. In the United States, a report by the Center for Disease Control and Prevention showed that 47.2% of adult people ≥30 years of age and 70.1% of the older people ≥65 years of age have some form of periodontal disease as their prevalence increases with age. Moreover, the condition is more common in men (56.4%) compared to women (38.4%) and is more prevalent in the population educated atbelow the high school level (66.9%) and in cigarette smokers (64.2%) [9]. 

Recent studies have recognized the emerging role of periodontitis in systemic inflammation and, in turn, in systemic disease states, such as oral cancer, Alzheimer’s disease, rheumatoid arthritis, diabetes, atherosclerosis, and inflammatory bowel disease [10]. Higher levels of systemic inflammation biomarkers, such as pro-inflammatory cytokines, i.e., tumor necrosis factor-alpha (TNF-α), interleukin (IL)-1, IL-6 and C-reactive protein, as a result of microbial translocation from periodontal lesions have been consistently observed in patients with periodontal disease [11,12]. However, despite considerable progress, the deeper relationships between the immune, inflammatory, infectious, and systemic features of periodontitis are not yet fully elucidated and are still under debate. 

The greater impact that periodontal disease exerts on host health has drawn attention to its prevention and treatment at the initial stages of disease [13], as it is known that a patient with gingivitis can return to a state of full health, while a patient with periodontitis will remain as such for life, even following successful therapy. Treatment involves good oral hygiene, professional tooth cleaning, and the use of antibiotics and periodontal surgery [13]. Antiseptic mouthwash containing chlorhexidine has been developed to strengthen the effects of an oral hygiene routine. However, one of the major concerns with the use of chlorohexidine is microbial resistance, particularly in case of *Acinetobacter baumannii*, *Escherichia coli*, methicillin-resistant *Staphylococcus aureus*, *Pseudomonas aeruginosa*, and *Klebsiella pneumoniae* [14,15]. The search for alternative, safe, and promising treatments for gingivitis represents an urgent need for the prevention of periodontitis and its systemic complications. In this context, traditional herbal medicines and plant-based food supplements could be considered as an alternative approach aimed at avoiding the possible development of periodontitis and the adverse effects of strains resistant to chlorhexidine [16,17,18].

*Cistus* × *incanus* L. belongs to the family Cistaceae and is widespread along the Mediterranean coast of Europe. It was used as an effective anti-inflammatory and skin protective plant agent in Mediterranean folk medicines. Moreover, the use of *C. incanus* tea to rinse the mouth contributes to the degradation of biofilm, a well-known virulence factor, and the prevention of biofilm-induced diseases by decreasing the load of associated bacteria [19]. In addition to these properties, the extracts obtained from some *Cistus* species with compositions similar to *C. incanus* extracts are known for their antibacterial activity against oral cavity pathogens and have been suggested as alternative natural antibacterial and antibiofilm components against oral infections [20]. *Scutellaria lateriflora* L., also known as American skullcap, belongs to the family Lamiaceae and is one of most widely used nervine agents in North American and Western herbal medicine. Traditionally, it has been used to promote a healthy menstrual cycle and to treat hysteria, anxiety, insomnia, delirium tremens, epilepsy, withdrawal from barbiturates and tranquilizers, bronchitis, diarrhea, dysentery, jaundice, hepatitis, hypertension, thrombosis, and tumors [21]. Moreover, *Scutellaria baicalensis* shows synergistic antibacterial effects against oral bacterial biofilms in combination with chlorhexidine [22].

The increasing microbial resistance to chlorhexidine calls for adecrease in its use and the discovery of new combinations of plant extracts, which can act synergistically, with high antibacterial and antibiofilm properties. On the basis of the above information, the aim of this studywas to continue the researchon *C. incanus* and to evaluate the anti-gingivitis properties of *S. lateriflora* extracts, both alone and in combination with one another. This was done with the final aim of providing scientific evidence for the development of a new food supplement based on botanical extracts that is able to act at the oral-cavity level to prevent against periodontal diseases and improve the health of the oral cavity. Thus, two commercial extracts obtained from *C. incanus* and *S. lateriflora* were chemically characterized, and their oral bioaccessibility after in vitro simulated oral digestion was determined. Then, their in vitro antimicrobial activity against *P. gingivalis*, antibiofilm activity, and ability to enhance the barrier function of a gingival keratinocyte model system and exert a protective effect against invasion by *P. gingivalis* were evaluated.

## 2. Materials and Methods

### 2.1. Chemicals and Reagents

One batch of commercial, dry, powdered hydroalcoholic extract of *C. incanus* (standardized to contain ≥18% of total polyphenols, and arabic gum as a carrier agent), and one batch of commercial, dry, powdered hydroalcoholic extract of *S. lateriflora* (standardized to contain ≥10% of baicalin, and maize maltodextrin as carrier agent) obtained from the aerial parts of the plants, were provided by EPO S.R.L. (Milan, Italy). All the compounds used for the in vitro simulated oral digestion process were purchased from Carlo Erba (Milan, Italy): potassium chloride (KCl), dihydrogen potassium phosphate (KH_2_PO_4_), sodium carbonate (NaHCO_3_), magnesium chloride (MgCl_2_), ammonium carbonate (NH_4_)_2_CO_3_, calcium chloride (CaCl_2_), sodium chloride (NaCl), hydrochloric acid (HCl), and sodium hydroxide (NaOH). From Sigma–Aldrich, Merck KGaA (Milan, Italy), α-Amylase from *Bacillus licheniformis*, formic acid solution (1 M), acetic acid solution (1M), water, methanol, acetonitrile (ACN) LC–MS grade, 3-(4,5-dimethylthiazol-2-yl)-2,5-diphenyl tetrazolium bromide (MTT), and dimethyl sulfoxide (DMSO) were purchased. All media and reagents for the cell culture were purchased from Gibco (Milan, Italy).

### 2.2. Chemical Characterization of C. incanus and S. lateriflora Extracts Using Reversed-Phase, Ultra-High-Performance Liquid Chromatography (RP-UHPLC) Coupled with a Q-Exactive Hybrid Quadrupole Orbitrap Mass Spectrometer

Stock solutions were prepared for the *C. incanus* and *S. lateriflora* extracts by accurately weighing 200 mg of extract and diluting them with a solution of 50:50 *v*/*v* acidified water (0.1% *v*/*v* formic acid) and methanol to a concentration of 10 mg/mL. From the stock solutions, 1 mL was taken and filtered prior to analysis (0.45 μm and 0.20 µm Minisart RC 4 membrane filters). The analysis wasperformed on a Thermo Ultimate RS 3000 paired online with a Q-Exactive hybrid quadrupole Orbitrap mass spectrometer (Thermo Fisher Scientific, Bremen, Germany) equipped with a heated electrospray ionization probe (HESI II). For the RP-UHPLC analysis, a Kinetex^®^ EVOTM 150 mm × 2.1 mm, 2.6 µm (L × I.D, particle size, Phenomenex^®^, Bologna, Italy) column was employed at a flow rate of 0.4 mL/min. The mobile phases consisted of (A) 0.1% CH_3_COOH in H_2_O and (B) ACN plus 0.1% CH_3_COOH. The analysis was performed in a gradient as follows: 0–10.0 min, 2–35% B; 10–12 min, 35–70% B; 12–13 min, 70–98% B; hold for 2 min; and return to initial conditions after 0.1 min. The column oven was set to 40 °C and 5 µL of the extracts were injected. An HRMS analysis was performed with Full MS (*m*/*z* 100–850) and data-dependent acquisition (dd-MS2 top; N = 5). The resolution selected was 70,000 and 15,000 FWHM at *m*/*z* 200. Stepped normalized collision energy (NCE) was used with values of 15, 25, and 30. The negative ion mode (ESI-) was employed. Source parameters were as follows: sheath gas pressure, 50 arbitrary units; auxiliary gas flow, 13 arbitrary units; spray voltage, −2.50 kV; capillary temperature, 260 °C; auxiliary gas heater temperature, 300 °C; and S-lens RF value, 30 arbitrary units. Metabolite annotation was performed using Compound Discoverer (Thermo Scientific, V3.3, Waltham, MA, USA) in comparison with in silico natural product libraries, accurate mass, and theexisting literature as previously reported [23].

### 2.3. In Vitro Bioaccessibility of C. incanus and S. lateriflora Extracts Using Simulated Oral Digestion Processes and RP-UHPLC-Photodiode Array Detector (PDA) Analysis

The current study aims to evaluate the possibility of a new food supplement for acting locally in the oral cavity against the pathogens associated with periodontal diseases; thus, the impact of the oral digestion process was verified on the chemical composition of *C. incanus* and *S. lateriflora* extracts following a protocol set by Minekus et al. with some modifications [24]. In brief, 5 g of each extract were dissolved in 3.5 mL of previously prepared, simulated salivary fluid (SSF) comprising an electrolyte solution containing (K^+^), (Na^+^), (Cl^−^), (H_2_PO_4_^−^), (HCO_3_^−^, CO_3_^2−^), (Mg^2+^), (NH_4_^+^), and (Ca^2+^). The same procedure was followed for the blank sample using 5 mL of water instead of the extracts. Then, 0.5 mL (1500 U/mL) of fresh α-amylase solution was added to both samples. In the end, water was added for the samples to reach a final volume of 10 mL, and the samples were incubated for 2 min at 37 °C. At the end of the oral digestion process, the samples were freeze dried and maintained at 4 °C prior to the RP-UHPLC-PDA analysis, which was performed on a Shimadzu Nexera LC30 (Shimadzu, Kyoto, Japan) with the same chromatographic conditions reported above; chromatograms were extracted at 280 and 330 nm.

### 2.4. Antimicrobial Activity of C. incanus and S. lateriflora Extracts against P. gingivalis

To evaluate the antimicrobial activity of *C. incanus* extract, *S. lateriflora* extract, their combinations in different ratios with the final concentrations of 60 mg/mL, and their carrier agents (i.e., maize maltodextrin and arabic gum), *P. gingivalis* (ATCC 33277), obtained from the LGC spa (ATCC distributor, Milan, Italy), was grown in a TSB-yeast extract medium supplemented with 0.05% cysteine hydrochloride, 0.02 μg/mL menadione, 5 μg/mL hemin, and 0.02% potassium nitrate in an anaerobic chamber (with 5% CO_2_) at 37 °C. In brief, serial dilutions of the samples were prepared at volumes of 100 μL/well in 96-well plates. The final concentrations of each of these were in the range of 60 to 5 mg/mL. To each well 20 μL of *P. gingivalis*, bacterial cell suspension was added at a final concentration of 1 × 10^6^ colony-forming units (CFU)/mL. Amoxicillin (10 µg/mL) was used as a positive control. After incubation in an anaerobic chamber at 37 °C for 24 h, bacterial growth was then analyzed using a microplate reader (Tecan, Männedorf, Swiss) at 595 nm. Each test was performed in triplicate. The rate of growth inhibition was determined using the following formula:

% Growth inhibition = 100 − [(100 × OD595 nm of the test sample)/OD595 nm of CTR]

### 2.5. In Vitro Cell Model Systems

#### 2.5.1. Human Keratinocyte Epithelial Cells (HaCaT)

Human immortalized keratinocytes (HaCaT) were grown as monolayers in a standard culture medium, Dulbecco’s Modified Eagle Medium (DMEM-10928_Gibco), and supplemented with 10% fetal bovine serum (FBS), 2 mM L-glutamine, 100 IU/mL penicillin, and 100 μg/mL streptomycin at 37 °C in a humidified atmosphere of 5% CO_2_ and 95% air. The medium was replaced every 48 h. The trypsinization process for HaCaT cells was always performed at 70% confluence.

#### 2.5.2. Cytotoxic Activity of *C. incanus* and *S. lateriflora* Extracts on HaCaT Cells

To assess the cytotoxic activity of the *C. incanus* and *S. lateriflora* extracts on HaCaT, alone and in combination, a 3-(4,5-dimethylthiazol-2-yl)-2,5-diphenyltetrazolium bromide (MTT) assay was performed. The MTT assay measures cellular metabolic activity as an indicator of vitality, proliferation, and cellular cytotoxicity [25]. Cells were cultured in DMEM and supplemented with 1% penicillin-streptomycin and 10% fetal bovine serum at 37 °C with 5% CO_2_ in a humid environment, as previously described. A density of 5× 10^4^ cells/well was seeded into 96-well plates and incubated for 24 h with (1) *C. incanus* extract at different concentrations ranging from 5 to 60 mg/mL, (2) *S. lateriflora* extract at different concentrations ranging from 5 to 60 mg/mL, or (3) their combinations in different ratios with a final concentration of 60 mg/mL. After 24 h of treatment, 100 μL of MTT solution (at a final concentration 0.5 mg/mL) was added to each well for 3 h at 37 °C. Then, the formazan crystals were solubilized by adding 100 μL of 100% DMSO to each well, and the viability rate was recorded at OD at 570 nm using a microplate reader (Tecan, Männedorf, Switzerland). Each test was performed in triplicate.

#### 2.5.3. In Vitro Effects of *C. incanus* and *S. lateriflora* Extracts on Invasive Capacity of *P. gingivalis* Targeting HaCaT Cells

To investigate the effectiveness of the test extracts, alone and in combination, on the invasive capacity of *P. gingivalis*, an invasion assay was performed as described elsewhere [26]. HaCaT cells were seeded into 24-well plates (1 × 10^5^ cells/well) and grown to ~70–80% confluence. The day after the pre-treatment experimental scheme, the cell monolayers were starved for 2 h in a DMEM-10928_Gibco medium without antibiotics and were treated with the samples at different concentrations ranging from 15 to 60 mg/mL for 1 at 37 °C. After preincubation with the samples, cells were infected with 1.5 × 10^8^ CFU/mL *P. gingivalis*. In a parallel co-treatment experimental scheme, bacteria and samples at the same concentrations reported above were incubated for 1 h and then used for the cell monolayer treatment. For both experimental schemes, after 4 h of infection, cells were washed with PBS three times and then incubated with gentamicin (Sigma–Aldrich, 100 μg/mL) to kill all extracellular bacteria. After 2 h in the presence of gentamicin, cells were lysed with 0.1% Triton-X solution to evaluate the amountof intracellular bacteria. Serial dilutions of the cell lysates were made in PBS, plated on TB agar, and incubated at 37 °C overnight. Then, the CFU/mL was counted relative to the bacteria that invaded the cell monolayer after incubation for 24 h at 37 °C.

#### 2.5.4. Effects of *C. incanus* and *S. lateriflora* Extracts on Pre-Formed Biofilm Mass Reduction

The ability for *C. incanus* and *S. lateriflora* extracts to degrade pre-formed biofilm was evaluated using acrystal violet (CV) assay [27]. Briefly, a bacterial inoculum was prepared at a density of 1 × 10^8^ CFU/mL in TSB supplemented with 1% glucose. A volume of 100 µL of bacterial suspension was transferred to each well of a 96-well plate and incubated at 37 °C for 24 h under static conditions to allow biofilm formation. After incubation, non-adherent cells were removed through PBS washes, and *C. incanus* and *S. lateriflora* extracts at different concentrations ranging from 5 to 55 mg/mL were added to the mature biofilm. The untreated and EDTA-treated biofilms constituted negative and positive controls, respectively. After treatment, the growth medium was removed, and the biofilm was gently washed with PBS. The biofilm biomass was quantified by adding 100 µL of 0.1% CV to each well for 30 min at room temperature under shaken conditions. Excess CV was removed, washed with PBS, and then solubilized with 98% ethanol for 40 min at room temperature under shaking conditions. Absorbance values recorded at 570 nm using a microplate reader (Tecan, Männedorf, Swiss) and were proportional to the biofilm mass present, and the results were expressed by calculating the percentage of reduction of the biofilm mass compared to the control samples.

### 2.6. Statistical Analysis

Data are reported as mean ± standard deviation (SD). The bacterial growth percentage and the cell invasive capacity were compared with the control sample at each examination point using an independent samples *t*-test, setting the level of significance at *p* < 0.05. Moreover, a statistical comparison among groups was conducted with multiple *t*-tests for multiple comparison using the Holm–Sidak method to analyze the bacterial growth inhibition percentage or the biofilm mass reduction percentage induced by the plant extract combinations to determine significance, which was set to *p* < 0.05. For each concentration used in both the antimicrobial and antibiofilm activities, a biological replicate was obtained and averaged. The statistical analyses were performed using GraphPad Prism, version 8 (San Diego, CA, USA).

## 3. Results

### 3.1. Metabolic Profile of C. incanus and S. lateriflora Extracts

**Figure 1:**
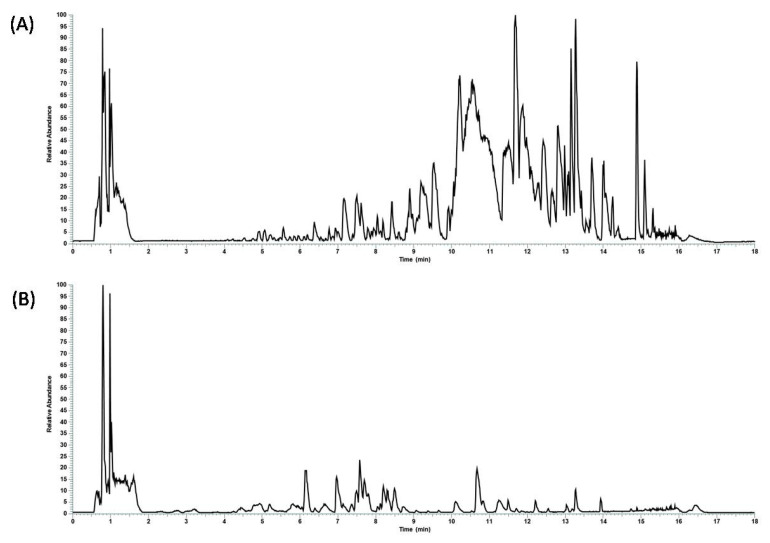
Base peak chromatograms revealing the metabolic profile of undigested (**A**) *S. lateriflora* and (**B**) *C. incanus* extracts.

The first step was the chemical characterization of the commercial hydroalcoholic *C. incanus* and *S. lateriflora* extracts using RP-UHPLC coupled with a Q Exactive hybrid quadrupole-Orbitrap mass spectrometer. Through a comparison with in silico MS/MS spectra, accurate mass, and molecular formula, 101 and 117 compounds were tentatively annotated in *C. incanus* and *S. lateriflora* extracts, respectively, with confidence MSI lvl.2 [28] as reported in the Supplementary Tables. The base peak chromatograms are reported in Figure 1.

### 3.2. Bioaccessibility of C. incanus and S. lateriflora Extracts after In Vitro Simulated Oral Digestion

**Figure 2:**
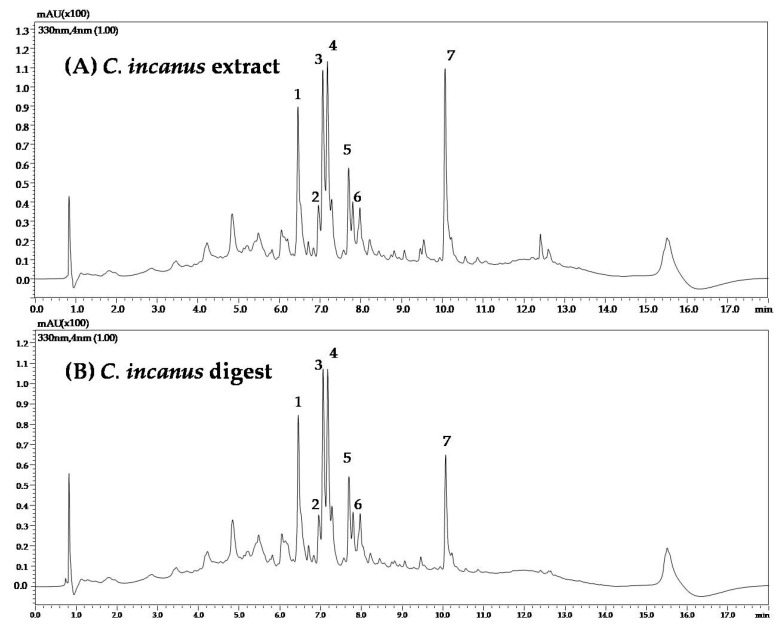
UV traces of *C. incanus* extract (**A**) before and (**B**) after in vitro simulated oral digestion.

**Figure 3:**
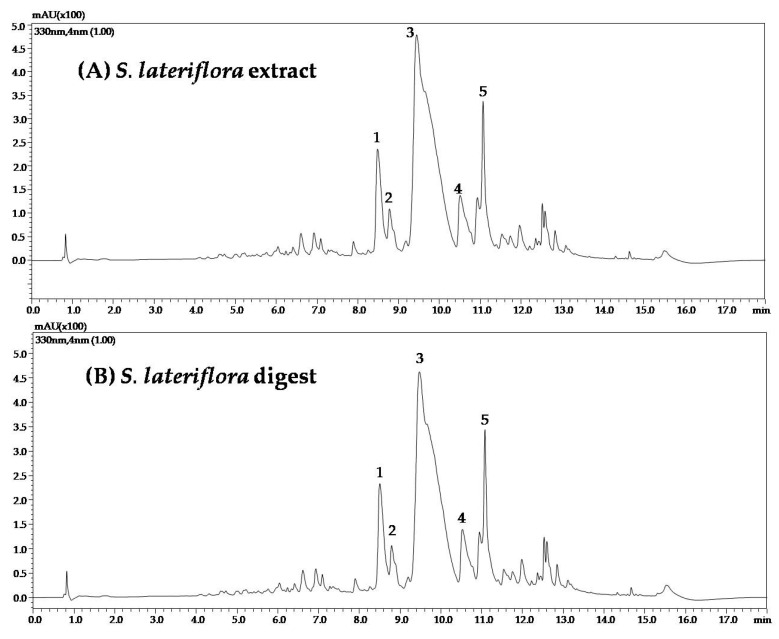
UV traces of *S. lateriflora* extract (**A**) before and (**B**) after in vitro simulated oral digestion.

To evaluate the influence of the in vitro simulated oral digestion process on the compounds most represented in *C. incanus* and *S. lateriflora* extracts, they were analyzed using RP-UHPLC-PDA before and after oral digestion. A slight shift in retention times was observed for orally digested samples, probably due to a matrix effect reduction following in vitro simulated oral digestion. UV traces of *C. incanus* and *S. lateriflora* extracts before and after in vitro simulated oral digestion are reported in Figure 2 and Figure 3, respectively.

Table 1 shows the mean peak area reduction percentages, ranging from 6.4 to 11.6%, after oral digestion of *C. incanus* extract, with the exception of kaempferol 3-(3″-*p*-coumaoroylhexoside), which was degraded by 46% when compared with the peak area recorded before digestion, revealing a moderate degradation process. Table 2 shows the mean peak area reduction percentage after oral digestion of *S. lateriflora* extract main peaks, which was found to be lower than 10%, revealing a modest degradation process.

### 3.3. Antibacterial Activity of C. incanus and S. lateriflora Extracts against P. gingivalis

**Figure 4:**
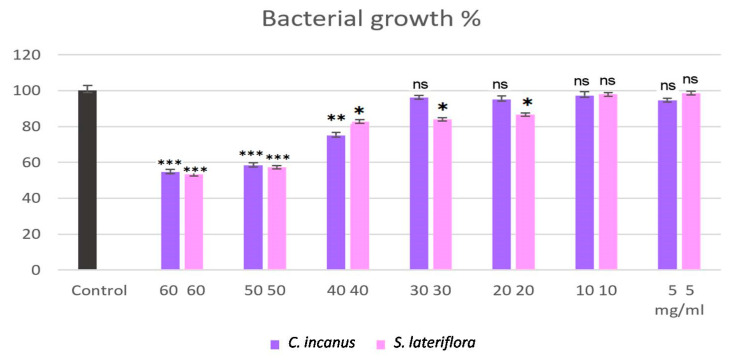
Bacterial growth percentage of *C. incanus* and *S. lateriflora* extracts at different concentrations against *P. gingivalis.* Results are expressed as mean ± SD from three biological replicates (*n* = 3). ns: *p* > 0.05, *: *p* < 0.05, **: *p* < 0.01, ***: *p* < 0.001.

*C. incanus* and *S. lateriflora* extracts were tested for their antibacterial activity against *P. gingivalis*. The results showed mild dose-dependent bacterial growth inhibitory activity, which did not allow for the determination of the minimum inhibitory concentration (MIC) values. In fact, at the highest concentration (60 mg/mL) at the end of the treatment, the percentage of *P. gingivalis* growth was found to be reduced to 55% and 53% for *C. incanus* and *S. lateriflora*, respectively (Figure 4), in comparison to the control sample, in which *P. gingivalis* was grown without the extracts. In particular, compared to the control sample, the statistically significant differences in the inhibition of microbial growth by *C. incanus* extract and *S. lateriflora* extract treatments were recorded starting from the concentration of 40 mg/mL (*p* = 0.0025) and 20 mg/mL (*p* = 0.0198), respectively (Figure 4).

As expected, neither maltodextrin nor arabic gum exerted inhibitory effects on the bacterial growth.

**Figure 5:**
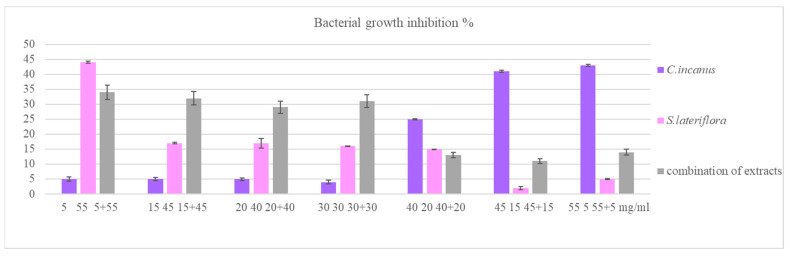
Bacterial growth inhibition percentage of *C. incanus* and *S. lateriflora* alone and in combinations at different concentrations against *P. gingivalis*.

Considering the low recorded antibacterial activity, the extracts used alone (at the concentrations of 5, 15, 20, and 30 mg/mL) and their combinations in different ratios (i.e., 1:1, 1:2, 1:3, etc.) with the final concentrations of 60 mg/mL were subjected to the same test. The results, reported in Figure 5, are expressed as percentage of bacterial growth inhibition. As regards the antimicrobial activities of *C. incanus* and *S. lateriflora* used alone, the results of the statistical analysis have already been reported in Figure 4. As regards the antimicrobial activities of *C. incanus* and *S. lateriflora* combinations, a comparison between the bacterial growth percentage recorded following the treatment with *C. incanus* and *S. lateriflora* combinations and the bacterial growth percentage recorded in their absence (control sample) shows a significance in every combination (*p* < 0.05), including those combinations in which *C. incanus* used alone at concentrations lower than 40 mg/mL was not found to be effective.

**Figure 6:**
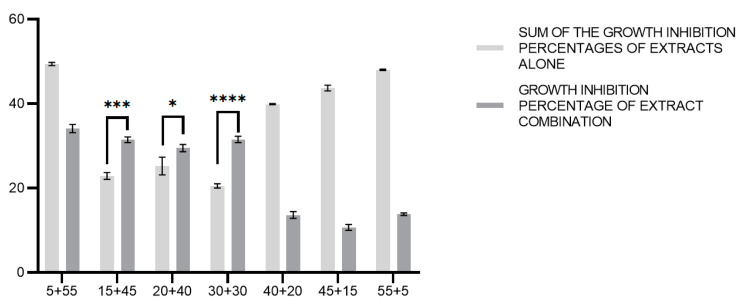
Comparison between the sums of the growth inhibition percentages of *C. incanus* and *S. lateriflora* alone and the growth inhibition percentages of *C. incanus* and *S. lateriflora* in combination. Results are expressed as mean ± SD from three biological replicated (*n* = 3). *: *p* < 0.05, ***: *p* < 0.001, ****: *p* < 0.0001.

Interestingly, the results show that, for the combinations of *C. incanus* and *S. lateriflora* at concentration ratios of 1:3, 1:2, and 1:1, the percentages of bacterial growth inhibition are greater than the sum of the percentages of bacterial growth inhibition recorded for the individual *C. incanus* and *S. lateriflora* extracts at the same concentrations. More specifically, we compared the sums of the bacterial growth inhibitory activities induced by the treatments with *C. incanus* and *S. lateriflora* extracts used alone with the bacterial growth inhibitory activities induced by the combinations of these extracts using a multiple *t*-test analysis. As regards the combinations of *C. incanus* and *S. lateriflora* (at concentration ratios of 1:3, 1:2, and 1:1), the differences between the sum of the activities of the individual extracts and the activities of these combinations was found to be statistically significant (*p* < 0.001, *p* < 0.05, and *p* < 0.0001, respectively), with the combinations of extracts showing higher activities. In contrast, in the other cases, the sum of the activities of the individual extracts was greater when compared to the effects shown by the combinations of the two extracts (Figure 6). 

### 3.4. Modulating Effects of C. incanus and S. lateriflora Extracts and Their Combinations on P. gingivalis Cell Invasive Capacity

To evaluate the activity of *C. incanus* and *S. lateriflora* extracts and their combinations on reducing the invasiveness of *P. gingivalis* in a HaCaT model system, HaCaT cells were treated with non-cytotoxic concentrations of *C. incanus* and *S. lateriflora* extracts (ranging from 10 to 60 mg/mL) before infection with *P. gingivalis*. The same was performed for the extract combinations at different ratios (i.e., 1:1, 1:2, 1:3, etc.) with final concentrations of 60 mg/mL (pre-treatment experimental condition). In addition, the co-treatment of HaCaT cells with *P. gingivalis* and the extracts used alone or in combination was also performed (co-treatment experimental condition). The results show that only the combination of *C. incanus* and *S. lateriflora* at the highest tested concentration (60 mg/mL), in the ratio 1:1, reduced the invasiveness of *P. gingivalis*, reflected as 6.4 × 10^5^ ± 1.1 × 10^4^ CFU/mL, compared to the control of 6.4 × 10^7^ ± 2.0 × 10^5^ CFU/mL (*p* = 0.0006). The other samples showed no modulation of the bacterial invasive capacity.

### 3.5. Effects of C. incanus and S. lateriflora Extracts and Their Combinations on the Degradation of Pre-Formed P. gingivalis Biofilm

**Figure 7:**
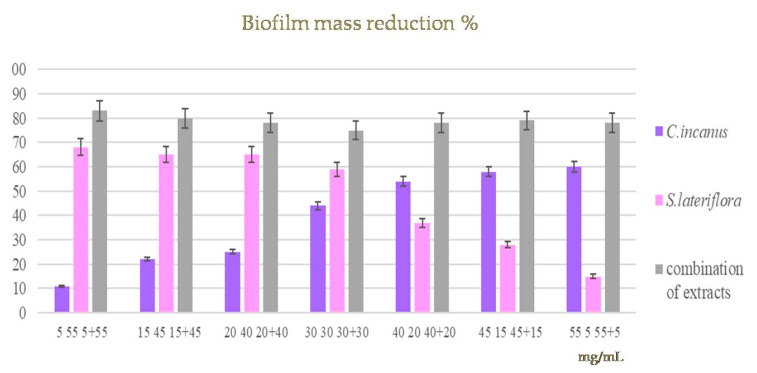
A comparison between the mass of biofilm produced by *P. gingivalis* following treatment with *C. incanus* and *S. lateriflora* extracts alone, in combination, and in their absence (control) showed a significance starting at the first concentration tested (5 mg/mL).

Biofilm formation is one of the main virulence mechanisms of *P. gingivalis*, contributing to an increase in the gingival tissue degradation process. The activity of *C. incanus* and *S. lateriflora* extracts and their combinations was evaluated on pre-formed biofilm. The biofilm biomass was quantified using CV in response to the treatments with the extracts used alone (at concentrations ranging from 5 to 55 mg/mL) and their combinations in different ratios (i.e., 1:1, 1:2, 1:3, etc.) with the final concentrations of 60 mg/mL, and then compared to measurements of the untreated mature biofilm. After 20 h of incubation, the samples induced a reduction in biofilm mass ranging from 11 to 56% and from 15 to 68% for the individual *C. incanus* and *S. lateriflora*, respectively. The combinations of the extracts in different ratios induced a biofilm mass reduction of about 80% (Figure 7). The statistical analysis shows that, by comparing the mass of biofilm produced by *P. gingivalis* following the treatment with *C. incanus* and *S. lateriflora* extracts used alone, in combination, and in their absence (control sample), we observed a statistically significant difference startingat the first tested concentration (5 mg/mL) (*p* < 0.05).

**Figure 8:**
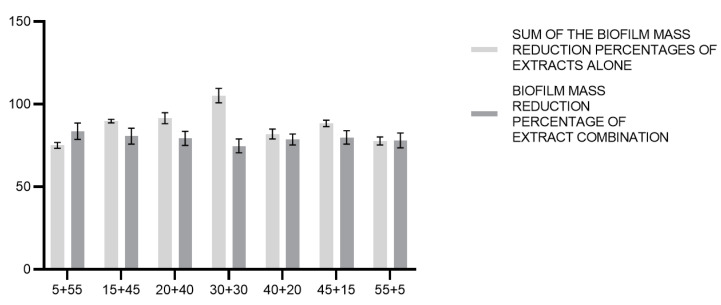
Comparison between the theoretical sum of the biofilm mass reduction percentages of *C. incanus* and *S. lateriflora* in combinations and the biofilm mass reduction percentage of extract combination.

As regards the percentage of biofilm mass reduction inhibition, the sums of the activities exerted by *C. incanus* and *S. lateriflora* extracts used alone and those of the extract combinations were compared through a multiple *t*-test analysis. The results showed that the sum of the activities exerted by *C. incanus* and *S. lateriflora* used alone were greater compared to the effects exerted by the combinations of the two extracts (Figure 8). In this case, no significant difference was demonstrated (*p* < 0.05).

## 4. Discussion

*P. gingivalis* is one of the major pathogens responsible for severe and chronic manifestations of periodontal disease, producing a number of virulence factors that cause direct and indirect destruction to periodontal tissues through modulation of the host inflammatory response [29]. Although human gingival epithelium prevents intrusion by periodontal bacteria, *P. gingivalis* is able to invade gingival epithelial cells. Without any doubt, primary prevention (including the use of toothbrushes, dental floss, water picks, toothpicks, small interproximal brushes, rubber gum stimulators, and mouthwash with antimicrobial and antibiofilm activities) is not only the most effective but also the cheapest way of coping with periodontitis and its complications [30]. Considering microbial resistance to the antibacterial agents, scientific research draws its attention towards the assessment of plant extracts (rich in bioactive phytochemicals, i.e., flavonoids, alkaloids, tannins, and terpenoids) with antimicrobial activity that may counteract emerging microbial resistance, while exhibiting antimicrobial properties against *P. gingivalis* [31]. In this study, we demonstrate that acombination of two commercial extracts obtained from *C. incanus* and *S. lateriflora*, which consists of a complex mixture of bioactive compounds that is stable after in vitro simulated oral digestion, decreases the in vitro growth of *P. gingivalis* and enhances the barrier function of a gingival keratinocyte model system, exerting both protective effects against invasion by *P. gingivalis* and antibiofilm activity. In more detail, as far as the phytochemical profiles of *C. incanus* and *S. lateriflora* extracts are concerned, more than one hundred compounds were identified for each extract. The results obtained support previous reports on the phytochemical compositions of *C. incanus* and *S. lateriflora* extracts, although to date no study has determined the metabolic profile of these extracts in such detail [21,32,33,34,35,36,37,38,39]. Moreover, the bioaccessibility of *C. incanus* and *S. lateriflora* polyphenolic compounds, to which their antibacterial and antibiofilm activities against periodontal pathogens are ascribed, has a strong impact on their ability to exert their biological activities in the oral cavity and was assessed. To the best of our knowledge, no earlier investigation has been published indicating the bioaccessibility of *C. incanus* and *S. lateriflora* polyphenols after oral digestion, and we are reporting for the first time that the concentrations of the most represented polyphenols occurring in *C. incanus* and *S. lateriflora* extracts are stable under oral digestion conditions.

As far as the antibacterial activities of *C. incanus* and *S. lateriflora* extracts against *P. gingivalis* are concerned, our results show a mild inhibitory effecton bacterial growth for both extracts in a dose-dependent manner. Interestingly, the combinations of the extracts exerted a greater inhibition of bacterial growth, especially when *S. lateriflora* is present in the culture medium in higher concentrations than those of *C. incanus*. To the best of our knowledge, the antimicrobial activities of *C. incanus* and *S. lateriflora* extracts alone and in combination against *P. gingivalis* have never been studied. These results could be considered in agreement with data from the existing literature that demonstrates the antimicrobial properties of *C. incanus* against Gram-positive pathogens (i.e., *S. aureus* and *S. epidermidis*) [40] and *Streptococcus mutans* colonization on enamel samples exposed to oral fluids [34]. Moreover, *S. lateriflora* root extract showed antibacterial effects against *Bacillus subtilis* (NCIMB 3610) and *E. coli* (NCIMB 8879) with a minimum inhibition concentration of 5 mg/mL and 25 mg/mL, respectively [41]. Moreover, the combination of *C. incanus* and *S. lateriflora* yielded a slight reduction in the cellular invasiveness of *P. gingivalis* at the highest tested concentration in pre-treatment assay conditions. To the best of our knowledge, no earlier investigation has been published on the reduction in the cellular invasiveness of *P. gingivalis* in the presence of *C. incanus* and *S. lateriflora* extracts. The invasion of host cells is the first step for bacteria to establish pathogenic reservoirs and evade host defense mechanisms [42]. Furthermore, while the human gingival epithelium prevents intrusion by periodontal bacteria, *P. gingivalis* is able to invade gingival epithelial cells (primarily through the action of *P. gingivalis* proteases), breaking the oral epithelial barrier and spreading into periodontal tissues [42]. Thus, although the reduction in invasiveness recorded is moderate, it could contribute to a reduction in infection and periodontal tissue injury. Finally, as regards the capability of *C. incanus* and *S. lateriflora* to modulate mature *P. gingivalis* biofilm, known as subgingival plaque, the results show that the combination of these extracts is capable of the almost total degradation of biofilm. This result is all the more important considering that bacteria within a biofilm have shown 10–1000 times more antibiotic resistance than planktonic bacteria [43]. Oral biofilm protects bacteria from counteraction by the host’s immune system and antibacterial agents in vivo. Furthermore, cells embedded in biofilm are more resistant to antibiotic treatment as the virulence factors produced by *P. gingivalis* are contained within the biofilm, such as fimbriae, hemagglutinins, and proteinases [44]. The results obtained support previous investigations showing the antibiofilm activity of the extracts obtained from different species of the *Cistus* genus (i.e., *C. creticus* L., *C. monspeliensis* L., and *C. laurifolius* L.) and *Scutellaria* genus (i.e., *S. baicalensis*) against biofilm formation of Gram-positive bacteria (*S. aureus*, *Bacillus subtilis*, and *S. mutans*) and Gram-negative bacteria (*E. coli*, *S. enterica*, *P. aeruginosa*, and *K. pneumonia*) [20,22,45,46].

In conclusion, the combined effects of *C. incanus* and *S. lateriflora* in the inhibition of the growth of *P. gingivalis* and its invasiveness as well asthe reduction of pre-formed biofilm mass may open considerations of their use in the treatment of gingivitis and as adjunctive therapeutic agents to periodontitis. Further in vitro studies reflecting insights into the mechanism of action and clinical trials assessing the efficacy of these extracts in human subjects are currently in progress.

## Figures and Tables

**Table 1:** Mean peak area of the main eight peaks identified in *C. incanus* extract before and after oral digestion and area reduction percentage (%).

*C. incanus* Compound	RT (min)	Mean Area before Digestion	Mean Area after Digestion	Area Reduction Percentage (%)
Myricetin 3-hexoside	6.45	3.66 × 10^5^	3.42× 10^5^	6.5
Myricetin 3 alpha L-arabinofuranoisde	6.95	1.05 × 10^5^	9.25 × 10^4^	11.6
Quercetin-3-*O*-glucopyranoside	7.06	3.93 × 10^5^	3.76 × 10^5^	4.3
Quercetin-3-*O*-glucopyranoside isomer	7.17	4.13 × 10^5^	3.75 × 10^5^	9.1
Gujaverin	7.69	1.54 × 10^5^	1.45 × 10^5^	6.4
Gujaverin isomer	7.79	7.40 × 10^4^	6.61 × 10^4^	10.6
Kaempferol 3-(3″-*p*-coumaoroylhexoside)	10.06	4.20 × 10^5^	2.27 × 10^5^	46.0

**Table 2:** Mean peak area of the main peak present in *S. lateriflora* extract after oral digestion and area reduction percentage (%).

*S. lateriflora* Compounds	RT (min)	Mean Area Phytcomplex	Mean Area Digest	Area Reduction Percentage (%)
Scutellarin	8.48	1.15 × 10^6^	1.05 × 10^6^	8.9
Isoscutellarin	8.78	4.66 × 10^5^	4.67 × 10^5^	0.0
Baicalein-6-glucuronide	9.44	3.01 × 10^7^	2.99 × 10^7^	1.0
Quercitrin	10.51	1.00 × 10^6^	9.32 × 10^5^	7.1
Oroxylin A-glucuronide	11.07	3.39 × 10^6^	3.39 × 10^6^	0.0

## Data Availability

Not available.

## Supplementary Materials

The following supporting information can be downloaded at: https://www.mdpi.com/article/10.3390/foods12091826/s1, Table S1. Identified compounds in C. incanus extract according to molecular formula, *m*/*z*, and the retention time (RT); Table S2. Identified compounds in S. lateriflora extract according to molecular formula, *m*/*z*, and the retention time (RT).
