# Supplementary material for: In Vitro Antimicrobial and Antibiofilm Properties and Bioaccessibility after Oral Digestion of Chemically Characterized Extracts Obtained from Cistus × incanus L., Scutellaria lateriflora L., and Their Combination
Source: Foods. 2023 Apr 28;12(9):1826. doi: 10.3390/foods12091826 (PMC10178160; doi:10.3390/foods12091826)
Supplement: Supplementary file 1 [file foods-12-01826-s001.zip › foods-2358610-SI.pdf]

foods

MDPI

Article

In vitro antimicrobial and antibiofilm properties and bioaccessibility after oral digestion of chemically characterized extracts obtained from *Cistus x incanus* L., *Scutellaria lateriflora* L., and their combination

Hammad Ullah[1,†], Alessandro Di Minno[1,2,†], Anna De Filippis[3], Eduardo Sommella[4], Daniele Giuseppe Buccato[1], Lorenza Francesca De Lellis[1], Hesham R. El-Seedi[5,6], Shaden A. M. Khalifa[7], Roberto Piccinocchi[8], Massimiliano Galdiero[3,9], Pietro Campiglia[4,10] and Maria Daglia[1,6,*]

Supplementary Tables

Table S1. Identified compounds in *C. incanus* extract according to molecular formula, *m/z*, and the retention time (RT)

Name	Formula	Δ [ppm]	*m/z*	RT [min]	Reference Ion
Punicalin	C34 H22 O22	1,04	781,0539	2,364	[M-H]-1
Gallic acid	C7 H6 O5	-4,5	169,0135	2,731	[M-H]-1
Galloca+ètechin	C15 H14 O7	0,52	305,0668	3,218	[M-H]-1
Gallocatechin isomer	C15 H14 O7	0,26	305,0668	4,718	[M-H]-1
Chlorogenic acid	C16 H18 O9	0,45	353,0880	4,732	[M-H]-1
Procyanidin B1	C30 H26 O12	0,26	577,1353	4,808	[M-H]-1
Caffeoyl-beta-D-hexose	C15 H18 O9	0,03	341,0878	4,924	[M-H]-1
Taxifolin 3-hexoside	C21 H22 O12	-0,06	465,1038	4,928	[M-H]-1
Corilagin	C27 H22 O18	0,33	633,0737	5,258	[M-H]-1
Syringic acid	C9 H10 O5	-3,16	197,0449	5,576	[M-H]-1
Chlorogenic acid isomer	C16 H18 O9	0,37	353,0879	5,596	[M-H]-1
6-Hydroxyluteolin 7-sophoroside	C27 H30 O17	0,53	625,1414	5,61	[M-H]-1
Procyanidin B1 isomer	C30 H26 O12	1,61	577,1361	5,687	[M-H]-1
Catechin	C15 H14 O6	0,9	289,0720	5,829	[M-H]-1
Catechin isomer	C15 H14 O6	-0,01	289,0718	5,86	[M-H]-1
Quercetin 3-alpha-L-arabinopyranosyl- (1->6) -hexoside	C26 H28 O16	1,21	595,1312	5,888	[M-H]-1
Chlorogenic acid isomer	C16 H18 O9	0,58	353,0880	6,048	[M-H]-1
Epigallocatechin 3-gallate	C22 H18 O11	0,43	457,0778	6,153	[M-H]-1
Myricetin 3-hexoside	C21 H20 O13	0,88	479,0835	6,558	[M-H]-1
Myricetin 3-sambubioside	C26 H28 O17	0,59	611,1257	6,612	[M-H]-1
Epigallocatechin 3-gallate isomer	C22 H18 O11	0,8	457,0780	6,621	[M-H]-1
Myricetin 3- (2''-galloylhexoside)	C28 H24 O17	0,55	631,0944	6,71	[M-H]-1
Quercetin 3,4'-dihexoside	C27 H30 O17	1,16	625,1418	6,753	[M-H]-1
Myricetin 3- (2''-galloylhexoside) isomer	C28 H24 O17	1,04	631,0947	6,928	[M-H]-1
Rhamnetin 3-hexoside isomer	C22 H22 O12	0,2	477,1039	6,964	[M-H]-1
Myricetin 3-hexoside isomer	C21 H20 O13	-0,28	479,0830	6,969	[M-H]-1

Rhamnetin 3-hexoside	C22 H22 O12	0,58	477,1041	6,999	[M-H]-1
Ferulic acid	C10 H10 O4	-3,59	193,0499	7,011	[M-H]-1
Quercetin 3- (2Gal-rhamnosyl-robinobioside)	C33 H40 O20	1	755,2048	7,038	[M-H]-1
Taxifolin 5-hexoside isomer	C21 H22 O12	0,36	465,1040	7,071	[M-H]-1
Quercetin-4'-hexoside	C21 H20 O12	0,65	463,0885	7,13	[M-H]-1
Phloretin 3',5'-Di-C-hexoside	C27 H34 O15	2,1	597,1838	7,153	[M-H]-1
Myricetin 3-alpha-L-arabinofuranoside	C20 H18 O12	0,76	449,0729	7,233	[M-H]-1
Quercetin 3-alpha-L-arabinopyranosyl- (1->6) -hexoside isomer	C26 H28 O16	0,88	595,1310	7,24	[M-H]-1
Taxifolin	C15 H12 O7	0,45	303,0512	7,277	[M-H]-1
Catechin 3-O-gallate	C22 H18 O10	0,17	441,0828	7,357	[M-H]-1
Catechin 3-O-gallate isomer	C22 H18 O10	0,51	441,0830	7,371	[M-H]-1
Catechin-4-ol 3'-methyl ether 3-O-alpha-L-rhamnopyranoside	C22 H26 O11	0,02	465,1402	7,419	[M-H]-1
Myricetin 3-alpha-L-arabinofuranoside isomer	C20 H18 O12	0,08	449,0726	7,481	[M-H]-1
Ellagic acid	C14 H6 O8	-0,3	300,9989	7,643	[M-H]-1
Quercetin-3-O-glucopyranoside (isoquercitrin) isomer	C21 H20 O12	0,21	463,0883	7,732	[M-H]-1
Myricetin 3-hexoside isomer	C21 H20 O13	0,6	479,0834	7,847	[M-H]-1
Kaempferol 7- (6''-galloylhexoside)	C28 H24 O15	1,03	599,1049	7,886	[M-H]-1
Myricetin	C15 H10 O8	0,81	317,0306	7,975	[M-H]-1
Dihydromyricetin	C15 H12 O8	0,96	319,0463	7,977	[M-H]-1
Isorhamnetin-O-hexoside	C22 H22 O12	0,64	477,1042	8,047	[M-H]-1
Guaijaverin isomer	C20 H18 O11	-0,14	433,0776	8,205	[M-H]-1
Quercetin 3-rhamnosyl- (1->6) - (2''-acetylhexoside)	C29 H32 O17	0,82	651,1572	8,229	[M-H]-1
Guaijaverin	C20 H18 O11	-0,28	433,0775	8,309	[M-H]-1
Guaijaverin isomer	C20 H18 O11	-0,03	433,0776	8,315	[M-H]-1
Dicaffoylquinic acid	C25 H24 O12	0,25	515,1196	8,387	[M-H]-1
Dihydrokaempferol	C15 H12 O6	0,73	287,0563	8,39	[M-H]-1
Quercetin-3-O-glucopyranoside (isoquercitrin) isomer	C21 H20 O12	0,61	463,0885	8,488	[M-H]-1
Trilobatin	C21 H24 O10	0,35	435,1298	8,61	[M-H]-1
Isorhamnetin-O-hexoside isomer	C22 H22 O12	0,51	477,1041	8,618	[M-H]-1
Rhamnocitrin 3-rutinoside	C28 H32 O15	1,56	607,1678	8,659	[M-H]-1
Kaempferol 3-alpha-L-arabinopyranoside isomer	C20 H18 O10	0,95	417,0831	8,737	[M-H]-1
Kaempferol 3-alpha-L-arabinopyranoside	C20 H18 O10	0,87	417,0831	8,74	[M-H]-1
Dicaffoylquinic acid isomer	C25 H24 O12	0,78	515,1199	8,819	[M-H]-1
Oleuropein	C25 H32 O13	0,88	539,1775	8,908	[M-H]-1
Quercetin-4'-hexoside isomer	C21 H20 O12	1,59	463,0889	8,942	[M-H]-1
Rosmarinic acid	C18 H16 O8	0,94	359,0776	9,07	[M-H]-1
Quercetin 3- (2G- (E) -p-coumaroylrutinoside)	C36 H36 O18	1,29	377,0881	9,098	[M-2H]-2
Quercetin 3- (6'''-p-coumarylglucosyl) (1->2) -rhamnoside	C36 H36 O18	1,86	755,1843	9,099	[M-H]-1

Quercetin	C15 H10 O7	0,82	301,0356	9,1	[M-H]-1
Myricetin 3- (6''-p-coumaroylhexoside)	C30 H26 O15	1,28	625,1207	9,131	[M-H]-1
Quercetin 3- (2''-galloyl-alpha-L-arabinopyranoside)	C27 H22 O15	1,05	585,0892	9,141	[M-H]-1
Kaempferol 3-glucuronide	C21 H18 O12	1,53	461,0733	9,262	[M-H]-1
Afzelin	C21 H20 O10	0,43	431,0986	9,3	[M-H]-1
Myricetin 3- (6''-p-coumaroylhexoside)	C30 H26 O15	1,05	625,1206	9,382	[M-H]-1
Quercetin 3- (2''-galloyl-alpha-L-arabinopyranoside) isomer	C27 H22 O15	0,99	585,0892	9,408	[M-H]-1
Quercetin 3- (6''-malonylhexoside)	C24 H22 O15	0,83	549,0891	9,428	[M-H]-1
Quercetin 3- (6''-acetylhexoside)	C23 H22 O13	0,98	505,0993	9,43	[M-H]-1
Kaempferol 3-glucuronide isomer	C21 H18 O12	1,26	461,0731	9,442	[M-H]-1
Quercetin 3-glucuronide	C21 H18 O13	0,98	477,0679	9,447	[M-H]-1
Daidzein	C15 H10 O4	0,29	253,0507	9,517	[M-H]-1
Dihydrokaempferol isomer	C15 H12 O6	0,83	287,0564	9,518	[M-H]-1
Quercetin 3- (6''-malonylhexoside) isomer	C24 H22 O15	1,17	549,0892	9,52	[M-H]-1
Chlorogenic acid isomer	C16 H18 O9	0,5	353,0880	9,619	[M-H]-1
Kaempferol 3-glucuronide isomer	C21 H18 O12	1,3	461,0732	9,724	[M-H]-1
Quercetin 3- (6''-malonylhexoside) isomer	C24 H22 O15	1	549,0892	9,751	[M-H]-1
Kaempferol 3- (6''-malonylhexoside)	C24 H22 O14	0,86	533,0941	9,983	[M-H]-1
Afzelin isomer	C21 H20 O10	0,61	431,0986	10,352	[M-H]-1
Isorhamnetin	C16 H12 O7	1,25	315,0514	10,371	[M-H]-1
Quercetin 3- (6''-malonylhexoside) isomer	C24 H22 O15	1,44	549,0894	10,435	[M-H]-1
Rhamnocitrin 3-hexoside	C22 H22 O11	1,64	461,1097	10,563	[M-H]-1
Kaempferol 3- (3''-p-coumarylhexoside)	C30 H26 O13	0,08	593,1301	10,667	[M-H]-1
Quercetin	C15 H10 O7	-0,13	301,0353	10,766	[M-H]-1
Naringenin	C15 H12 O5	0,3	271,0613	10,822	[M-H]-1
Kaempferol 3- (3''-p-coumarylhexoside) isomer	C30 H26 O13	0,37	593,1303	10,829	[M-H]-1
Kaempferol 3-glucuronide isomer	C21 H18 O12	1,47	461,0732	11,042	[M-H]-1
Genistein	C15 H10 O5	0,96	269,0458	11,094	[M-H]-1
Kaempferol	C15 H10 O6	1,03	285,0408	11,475	[M-H]-1
Kaempferol 3- (3''-p-coumarylhexoside) isomer	C30 H26 O13	1,22	593,1308	12,059	[M-H]-1
Hesperetin	C16 H14 O6	0,77	301,0720	12,112	[M-H]-1
Formononetin	C16 H12 O4	0,52	267,0664	12,254	[M-H]-1
Quercetin 3-methyl ether	C16 H12 O7	0,94	315,0513	12,5	[M-H]-1
Kaempferol 3- (3''-p-coumarylhexoside) isomer	C30 H26 O13	0,93	593,1306	12,57	[M-H]-1
Myricetin 3,3',5'-trimethyl ether	C18 H16 O8	1,19	359,0777	12,651	[M-H]-1
Kaempferol 3- (2'',6''-di- (E) -p-coumarylhexoside)	C39 H32 O15	0,99	739,1680	12,805	[M-H]-1
Kaempferol 3- (3'',6''-di-p-coumarylhexoside)	C39 H32 O15	1,09	739,1678	13,039	[M-H]-1

Table S2. Identified compounds in *S. lateriflora* extract according to molecular formula, *m/z*, and the retention time (RT)

**Name**	**Formula**	**Δ [ppm]**	***m/z***	**RT [min]**	**Reference Ion**
Gallic acid	C7 H6 O5	-4,59	169,0135	1,588	[M-H]-1
Chlorogenic acid	C16 H18 O9	0,76	353,0881	4,76	[M-H]-1
Chlorogenic acid dimer	C32 H36 O18	2,06	707,1844	5,597	[M-H]-1
Chlorogenic acid isomer	C16 H18 O9	0,41	353,0880	5,609	[M-H]-1
Chlorogenic acid isomer	C16 H18 O9	0,6	353,0880	6,059	[M-H]-1
Kaempferol-3-O-hexo rhamnoside isomer	C27 H30 O15	0,96	593,1518	6,312	[M-H]-1
Carthamidin 7-O-glucoronide	C21 H20 O12	1,18	463,0887	6,676	[M-H]-1
Ferulic acid	C10 H10 O4	-3,16	193,0500	6,733	[M-H]-1
Pentahydroxyflavanone	C21 H22 O11	0,64	449,1092	6,846	[M-H]-1
Schaftoside	C26 H28 O14	0,48	563,1409	6,865	[M-H]-1
Quercetin-dihexoside	C27 H30 O17	0,74	625,1415	6,965	[M-H]-1
Myricetin-hexoside	C21 H20 O13	0,12	479,0832	6,966	[M-H]-1
Luteolin 7,3'-diglucuronide	C27 H26 O18	0,87	637,1052	7,011	[M-H]-1
Quercetin-hexoside	C21 H20 O12	0,56	463,0885	7,129	[M-H]-1
6-C-Glucopyranosyldihydrokaempferol	C21 H22 O11	0,95	449,1094	7,222	[M-H]-1
Taxifolin	C15 H12 O7	0,32	303,0512	7,272	[M-H]-1
Quercetin 3-glucuronide	C21 H18 O13	0,79	477,0678	7,353	[M-H]-1
Schaftoside isomer	C26 H28 O14	0,53	563,1409	7,39	[M-H]-1
Luteolin 7-glucuronide	C21 H18 O12	0,98	461,0730	7,532	[M-H]-1
Ellagic acid	C14 H6 O8	0,38	300,9991	7,592	[M-H]-1
Rutin	C27 H30 O16	0,21	609,1462	7,595	[M-H]-1
6-C-beta-D-Hexosyl apigenin	C21 H20 O10	-0,27	431,0983	7,619	[M-H]-1
Kaempferol-3-O-hexo rhamnoside	C27 H30 O15	0,5	593,1515	7,642	[M-H]-1
Verbascoside	C29 H36 O15	0,65	623,1986	7,669	[M-H]-1
Quercetin-3-O-glucopyranoside (isoquercitrin)	C21 H20 O12	0,82	463,0886	7,686	[M-H]-1
Persicogenin-hexoside	C23 H26 O11	0,66	477,1406	7,754	[M-H]-1
Ferulic acid isomer	C10 H10 O4	-3,44	193,0500	7,762	[M-H]-1
Quercetin-hexoside isomer	C21 H20 O12	0,72	463,0885	7,789	[M-H]-1
Kaempferol 3-hexoside-7-glucuronide	C27 H28 O17	0,38	623,1256	7,849	[M-H]-1
Chrysin 8-C-β-d-hexoside	C21 H20 O9	0,29	415,1036	7,945	[M-H]-1
Ferulic acid isomer	C10 H10 O4	-3,63	193,0499	7,951	[M-H]-1
Quercetin 4'-methyl ether 3-hexoside	C22 H22 O12	0	477,1039	8,092	[M-H]-1
Kaempferol-3-O-hexo rhamnoside isomer	C27 H30 O15	0,7	593,1516	8,25	[M-H]-1
Isoorientin 3'-O-glucuronide	C27 H28 O17	0,48	623,1257	8,337	[M-H]-1
Dihydrokaempferol	C15 H12 O6	0,46	287,0562	8,382	[M-H]-1
Hesperetin 7-O-hexoside	C22 H24 O11	1,35	463,1252	8,384	[M-H]-1
Scutellarein 7,4'-dimethyl ether 6-sophoroside	C29 H34 O16	0,43	637,1777	8,442	[M-H]-1
Luteolin 3',4'-diglucuronide	C27 H26 O18	0,37	318,0487	8,471	[M-2H]-2

Persicogenin-hexoside isomer	C23 H26 O11	0,15	477,1403	8,487	[M-H]-1
Apigenin 7-glucuronosyl-hexoside	C27 H28 O16	1,06	607,1311	8,495	[M-H]-1
Isorhamnetin-O-hexoside	C22 H22 O12	0,17	477,1039	8,497	[M-H]-1
Rhamnetin 3-galactoside	C22 H22 O12	0,6	477,1041	8,498	[M-H]-1
Quercetin 3-rhamnosyl- (1->2) –hexoside	C27 H30 O16	0,87	609,1466	8,553	[M-H]-1
Myricetin 3-glucuronide	C21 H18 O14	0,99	493,0629	8,604	[M-H]-1
Dicaffeoylquiniq acid isomer	C25 H24 O12	0,78	515,1199	8,688	[M-H]-1
Cistanoside C	C30 H38 O15	1,5	637,2148	8,75	[M-H]-1
Trihydroxytrimethoxyflavanone	C22 H22 O12	0,42	477,1041	8,776	[M-H]-1
Rhamnocitrin 3-galactoside	C22 H22 O11	0,85	461,1093	8,806	[M-H]-1
Quercetin 4'-methyl ether 3-hexoside isomer	C22 H22 O12	0,19	477,1039	8,835	[M-H]-1
Eriodictyol 7-glucuronide	C21 H20 O12	0,65	463,0885	8,857	[M-H]-1
Quercetin 3-(6''-acetylhexoside)	C23 H22 O13	0,87	505,0992	8,899	[M-H]-1
Dicaffeoylquinic acid	C25 H24 O12	0,19	515,1196	8,926	[M-H]-1
Carthamidin 7-O-glucoronide isomer	C21 H20 O12	0,71	463,0885	8,964	[M-H]-1
Scutellarein 7,4'-dimethyl ether 6-sophoroside isomer	C29 H34 O16	1,28	637,1782	8,974	[M-H]-1
Quercitrin	C21 H20 O11	0,79	447,0936	8,978	[M-H]-1
Luteolin 3'-methyl ether 7-hexoside	C22 H22 O11	-0,04	461,1089	8,991	[M-H]-1
Rosmarinic acid	C18 H16 O8	0,59	359,0775	9,007	[M-H]-1
Apigenin 7,4'-diglucuronide	C27 H26 O17	0,64	621,1101	9,065	[M-H]-1
Orientin 2''-O-caffeate	C30 H26 O14	0,53	609,1253	9,099	[M-H]-1
Scutellarin	C21 H18 O12	0,38	461,0727	9,193	[M-H]-1
Kaempferol 3-rhamnosyl- (1->3) (4'''-acetylrhamnosyl) (1->6) -hexoside	C35 H42 O20	1,08	781,2205	9,194	[M-H]-1
Chrysin 8-C-β-d-hexoside isomer	C21 H20 O9	0,04	415,1035	9,233	[M-H]-1
Apigenin 8-C-hexoside	C21 H20 O10	-0,46	431,0982	9,274	[M-H]-1
Dicaffeoylquinic acid isomer	C25 H24 O12	0,37	515,1197	9,335	[M-H]-1
Dicaffeoylquiniq acid isomer	C25 H24 O12	0,26	515,1196	9,473	[M-H]-1
Isorhamnetin 3-glucuronide	C22 H20 O13	0,43	491,0833	9,479	[M-H]-1
Kaempferol 3- (6''-acetylhexoside) -7-rhamnoside	C29 H32 O16	0,65	635,1622	9,511	[M-H]-1
6-Hydroxyluteolin 6-glucuronide	C21 H18 O13	0,52	459,0572	9,529	[M-H-H2O]-1
Isorhamnetin 3-glucuronide isomer	C22 H20 O13	0,28	491,0833	9,624	[M-H]-1
Pinostrobin 5-O-glucoside	C22 H24 O9	0,37	431,1349	9,717	[M-H]-1
Norwogonin	C22 H22 O10	0,77	445,1144	9,735	[M-H]-1
2''-O-Feruloylorientin	C31 H28 O14	1,2	623,1414	9,801	[M-H]-1
2''-trans-Caffeoylisoorientin	C30 H26 O14	0,64	609,1254	9,815	[M-H]-1
Quercetin 7-glucuronide	C21 H18 O13	0,82	477,0679	9,862	[M-H]-1
Quercetin 7-xyloside	C20 H18 O11	1,27	433,0782	9,87	[M-H]-1
Lutonarin (Isoorientin 7-glucoside)	C27 H30 O16	0,92	609,1467	9,892	[M-H]-1
Baicalein 6-glucuronide	C21 H18 O11	0,42	445,0778	9,916	[M-H]-1
Isorhamnetin 3-glucuronide	C22 H20 O13	0,81	491,0835	9,945	[M-H]-1

Apigenin 7,4'-diglucuronide isomer	C27 H26 O17	0,92	621,1103	9,963	[M-H]-1
Pinosylvin	C14 H12 O2	-4,3	211,0755	10,097	[M-H]-1
5,8,3',4'-Tetrahydroxy-6,7-dimethoxyflavone	C17 H14 O8	-0,2	345,0615	10,14	[M-H]-1
Oroxylin A	C16 H12 O5	0,29	283,0613	10,159	[M-H]-1
Kaempferol 3- (4'',6''-diacetylhexoside) -7-rhamnoside isomer	C31 H34 O17	1,35	677,1732	10,196	[M-H]-1
Kaempferol	C15 H10 O6	0,6	285,0406	10,237	[M-H]-1
Vitexin	C21 H20 O10	0,64	491,1197	10,426	[M-H+HAc]-1
Kaempferol 3- (4'',6''-diacetylhexoside) -7-rhamnoside isomer	C31 H34 O17	1,32	677,1732	10,671	[M-H]-1
Kaempferol 3- (4'',6''-diacetylhexoside) -7-rhamnoside	C31 H34 O17	0,63	677,1728	10,687	[M-H]-1
Quercitrin isomer	C21 H20 O11	0,9	447,0937	10,798	[M-H]-1
Naringenin	C15 H12 O5	1,03	271,0615	10,832	[M-H]-1
Dihydrohydimethoxyflavanone	C23 H22 O12	0,28	489,1040	10,972	[M-H]-1
Apigenin 8-C- (6''-acetyhexoside)	C23 H22 O11	1,25	473,1095	11,148	[M-H]-1
Baicalein 6-methyl ether 7-glucuronide	C22 H20 O11	0,45	459,0935	11,294	[M-H]-1
Luteolin 7-lactate	C18 H14 O8	0,03	357,0616	11,466	[M-H]-1
Diihydroxynorwogonin	C15 H12 O5	0,75	271,0614	11,483	[M-H]-1
Isorhamnetin	C16 H12 O7	1,58	315,0515	11,695	[M-H]-1
Baicalein 6-glucuronide	C21 H18 O11	-0,03	445,0776	11,703	[M-H]-1
Apigenin 7-lactate	C18 H14 O7	-0,06	341,0667	11,716	[M-H]-1
Naringenin 7-O-beta-D-hexoside 6''-acetate	C23 H24 O11	0,78	475,1250	11,739	[M-H]-1
Oroxylin A glucuronide	C22 H20 O11	-0,18	459,0932	11,86	[M-H]-1
Wogonin	C16 H12 O5	-0,69	283,0610	11,867	[M-H]-1
Tenaxin II	C16 H12 O6	-0,83	299,0559	11,936	[M-H]-1
Viscidulin II	C17 H14 O7	0,14	311,0562	12,032	[M-H-H2O]-1
Quercitrin	C21 H20 O11	0,6	447,0936	12,332	[M-H]-1
Wogonin isomer	C16 H12 O5	-0,54	283,0610	12,428	[M-H]-1
Oroxylin A glucuronide isomer	C22 H20 O11	0,25	459,0934	12,43	[M-H]-1
Pinosylvin	C14 H12 O2	-2,51	211,0759	12,435	[M-H]-1
Apigenin 7-lactate isomer	C18 H14 O7	0,28	341,0668	12,525	[M-H]-1
Oroxylin A glucuronide	C22 H20 O11	0,64	459,0936	12,98	[M-H]-1
Pinocembrin	C15 H12 O4	-0,64	255,0661	13,045	[M-H]-1
Chrysin	C15 H10 O4	-0,03	253,0506	13,061	[M-H]-1
Baicalein 6-glucuronide	C21 H18 O11	0,62	445,0779	13,166	[M-H]-1
Oroxylin A glucuronide isomer	C22 H20 O11	0,7	459,0936	13,169	[M-H]-1
Scutellarein 7-glucuronide-6-ferulate	C31 H26 O15	1,4	637,1208	13,599	[M-H]-1
Chrysin 7-glucuronide	C21 H18 O10	0,28	429,0828	14,405	[M-H]-1
Pinocembrin isomer	C15 H12 O4	0,04	255,0663	14,458	[M-H]-1
Baicalein 6-glucuronide	C21 H18 O11	-0,18	445,0775	14,881	[M-H]-1
Genistein	C15 H10 O5	1,08	269,0458	16,047	[M-H]-1
